# Clinical on-site monitoring of ß-lactam antibiotics for a personalized antibiotherapy

**DOI:** 10.1038/s41598-017-03338-z

**Published:** 2017-06-09

**Authors:** R. Bruch, C. Chatelle, A. Kling, B. Rebmann, S. Wirth, S. Schumann, W. Weber, C. Dincer, G. Urban

**Affiliations:** 1 Department of Microsystems Engineering, University of Freiburg, 79110 Freiburg, Germany; 2 Faculty of Biology, University of Freiburg, 79104 Freiburg, Germany; 3 Department of Biosystems Science and Engineering, ETH Zurich, 4058 Basel, Switzerland; 4 Department of Anaesthesiology and Critical Care, Medical Center – University of Freiburg, Faculty of Medicine, University of Freiburg, 79106 Freiburg, Germany; 5 Freiburg Materials Research Center, University of Freiburg, 79104 Freiburg, Germany; 6 BIOSS Centre for Biological Signalling Studies, University of Freiburg, 79104 Freiburg, Germany

## Abstract

An appropriate antibiotherapy is crucial for the safety and recovery of patients. Depending on the clinical conditions of patients, the required dose to effectively eradicate an infection may vary. An inadequate dosing not only reduces the efficacy of the antibiotic, but also promotes the emergence of antimicrobial resistances. Therefore, a personalized therapy is of great interest for improved patients’ outcome and will reduce in long-term the prevalence of multidrug-resistances. In this context, on-site monitoring of the antibiotic blood concentration is fundamental to facilitate an individual adjustment of the antibiotherapy. Herein, we present a bioinspired approach for the bedside monitoring of free accessible ß-lactam antibiotics, including penicillins (piperacillin) and cephalosporins (cefuroxime and cefazolin) in untreated plasma samples. The introduced system combines a disposable microfluidic chip with a naturally occurring penicillin-binding protein, resulting in a high-performance platform, capable of gauging very low antibiotic concentrations (less than 6 ng ml^−1^) from only 1 µl of serum. The system’s applicability to a personalized antibiotherapy was successfully demonstrated by monitoring the pharmacokinetics of patients, treated with ß-lactam antibiotics, undergoing surgery.

## Introduction

Antibiotherapy is a central issue within the critical care medicine and the perioperative prophylactic treatment. Mainly, pneumonia and sepsis developing from an initial infection are major problems in the intensive care unit (ICU). Several approaches for the treatment of such infections are therefore in the focus of current research. Particularly, the appropriateness of the antibiotherapy was associated with improved survival^1^ and it could be shown that early applications of antibiotics are beneficial for patients^2^. Continuous drug infusion is currently discussed, however, no clear benefits could be demonstrated so far^3, 4^.

In this context, the adequate antibiotic dosage plays a crucial role in the efficacy of the anti-infective therapy. Antibiotic overdosing may increase the risk for toxic side effects without additional anti-infective benefits^5^. In contrast, too low doses may critically impair therapy^6^ and may promote the emergence of multidrug-resistant bacteria^7^. Today, antibiotics are dosed with regard to the body weight of the patient. Thereby, patient’s individual aspects, e.g. their physical status, severity of illness and thus, their metabolism, are however not considered. This frequently results in inadequate dosage^8, 9^. Therefore, an individualized antibiotherapy appears as a promising approach for the treatment of bacterial infections^10^. To appropriately adjust the dosage of the antibiotherapy, the patient’s personal pharmacokinetic must be determined. Moreover, monitoring the blood antibiotic concentration at the bedside would enable a quicker adjustment of the treatment. However, this is currently not part of the clinical routine, neither in the operating room (OR) nor in the ICU. The main limitation remains the lack of reliable, fast and cheap ways for the on-site quantification of antibiotics in clinically relevant specimens. Such an approach would enable the guidance for a personalized antibiotherapy, while decreasing the patient’s recovery time (e.g. in case of an under-dosed antibiotherapy).

Miniaturized diagnostic platforms offer an acceleration of the specific and sensitive detection of different analytes (e.g. biomarkers, hormones and drugs). Hence, they can be adapted to many areas of medicine and basic science. Herein, an attractive approach is a microfluidic lab-on-a-Chip (LoC) system, enabling device-portability, a low sample/reagent consumption and an easy integration of complex laboratory functions. Furthermore, the application of microfluidics with its advantageous surface-to-volume ratio leads to significantly faster sample-to-result times for the diagnostics compared to other tools like the microplate technology^11, 12^. Electrochemical sensors provide a high potential of miniaturization, have a low power consumption, are highly sensitive and are therefore the favourable technology for miniaturized point-of-care biosensors^13–15^. Such a microfluidic LoC device combined with a performant biomolecular sensing system could assure an adequate profiling of blood antibiotic levels and thus, prevent over- or under-dosing of antibiotics. This would improve the patients’ outcome and in long-term limit the further development of multi-drug resistant bacteria.

For a fast and low-cost detection of antibiotics, some naturally occurring biomolecules have emerged as biosensor with analytical properties hardly achieved by other bio-recognition methods (e.g. specific antibodies^16^): (i) they respond to a specific class of analytes diluted within a complex matrix (e.g. blood) and (ii) they show an inherent sensitivity to metabolite concentrations within the physiological concentration range^17^. For example, bacteria have evolved a class of regulator proteins responsive to antibiotic drugs like tetracycline, macrolide or streptogramin antibiotics, which was previously demonstrated successfully for the development of bioassays^16, 18^. In the same way, other bacterial proteins, which are known to be involved in the resistance building mechanism, can be implemented as biosensors. Those biomolecular sensors need then to be integrated into a diagnostic platform, combined with a readout technology, preferably compatible with point-of-care testing (POCT)^19^.

The class of ß-lactams belong to broad-spectrum antibiotics composed of penicillins, cephalosporins and carbapenems, all containing a ß-lactam ring in their chemical structure. This structure inhibits bacterial cell wall biosynthesis via the binding to key membrane proteins, so-called penicillin-binding proteins (PBPs)^20, 21^. The PBP3 from *S. pneumoniae* shows a high specificity and affinity towards the ring of most β-lactam antibiotics^21^. Moreover, a soluble truncated version of this protein has been conveniently overexpressed and purified with high yields in *E. coli* ^22^. Given these properties, it appears to be an attractive sensing biomolecule for the broadband quantification of ß-lactam antibiotics. In this context, we introduce here a disposable, electrochemical, microfluidic platform for bedside monitoring of various ß-lactam antibiotics in plasma samples, by implementing the PBP3 into an enzyme-linked assay (ELA) as a biomolecular sensor^22^. The systems’ applicability for a personalized antibiotherapy was successfully demonstrated by monitoring the individual pharmacokinetics of two patients, treated with commonly used cephalosporins, including cefuroxime and cefazolin, undergoing antibiotherapy during surgery.

## Results

### Design of a competitive bioassay for the quantification of ß-lactam antibiotics

**Figure 1:**
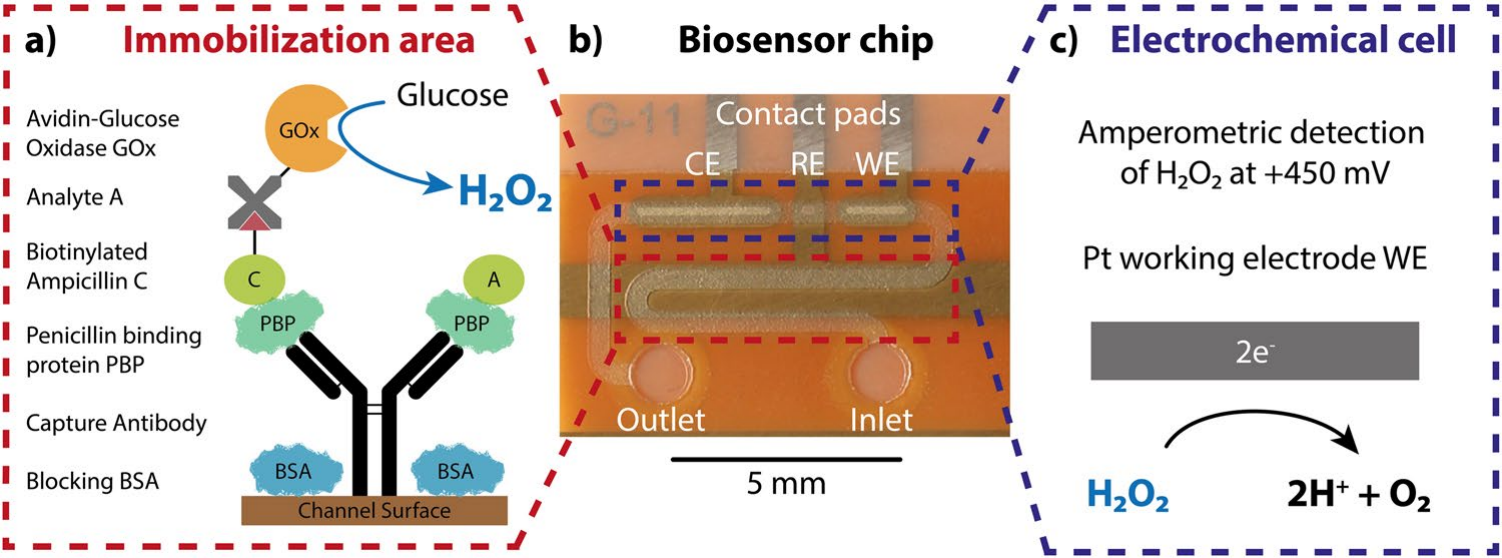
Illustration of the operating principle of the electrochemical microfluidic platform: (**a**) Schematic sketch of the competitive. ß-lactam assay, based on the PBP3 receptor. (**b**) Photograph of the microfluidic biosensor, showing its main elements, including the counter electrode CE, the reference electrode RE and the working electrode WE. The immobilization area is highlighted in red and the. electrochemical cell is marked in blue. (**c**) Schematic reaction of the oxidation of the produced hydrogen peroxide at the Pt working electrode for amperometric detection inside the electrochemical cell.

Based on the PBP3 from *S*. *pneumoniae*, a competitive bioassay for the quantification of β-lactam antibiotics has been designed and developed (Fig. 1a). It relies on the competitive binding of the analyte (β-lactam present in the sample of interest) and an ampicillin-biotin conjugate to the immobilized PBP3 receptor. The detection of the bound conjugate is performed via an enzyme (glucose oxidase, GOx) coupled to avidin and thus, specifically binding to the biotin from the conjugate. In presence of its substrate (glucose solution), the enzyme produces hydrogen peroxide (H_2_O_2_), which is electrochemically detected. The more analyte is present in the sample, the less conjugate is able to bind to the PBP3. Consequently, the strength of the signal is inversely proportional to the analyte concentration. Recombinant His-tagged PBP3 (Gly15-Lys394) was produced and purified from *E. coli*. Three different ampicillin-biotin conjugates with increasing linker length between the ampicillin and the biotin were chemically synthetized.

Preliminary tests revealed that the absence of a linker between the ampicillin and the biotin (conjugate 1) leads to a very low overall signal, probably due to a poor accessibility of the biotin to the enzyme avidin-GOx. This steric hindrance could be overcome by the use of conjugates with longer linkers. Conjugate 3 (ampicillin-PEG24-biotin), with the longest linker, was selected for further optimization as it resulted in an high overall assay signal, along with good sensitivity (Supplementary Figure S1). The functionality of each component of the assay, was tested and confirmed via the optical quantification of ampicillin on a microtiter plate (Supplementary Figure S2), before being transferred to the microfluidic platform.

### Working principle of the microfluidic biosensor

The microfluidic electrochemical biosensor employed in this work was manufactured using the dry film photoresist (DFR) technology^23–25^. It requires very low sample and reagent volumes (580 nl) and is mainly produced under standard laboratory conditions, reducing expensive clean-room processing. The biosensor chip consists of one single microfluidic channel, which comprises thereby two distinct areas: the immobilization area, where the bioassay is immobilized and the electrochemical cell for the readout of the assay signal (Fig. 1b). These two regions are separated by a hydrophobic stopping barrier, which allows a passively and precise metering of the fluids by capillary filling. It also prohibits an overflowing of reagents into the electrochemical readout cell, where otherwise electrode fouling would occur. As a signal transducer, GOx was used. The H_2_O_2_, produced by the enzyme, can then be oxidized at the platinum working electrode, releasing electrons, which generates a current signal, measured by means of amperometry (Fig. 1c). Under a constant glucose substrate flow through the channel, the bound GOx generate a certain amount of H_2_O_2_. To obtain a signal amplification along with a fast detection, the so called stop-flow technique has been applied^26^. By stopping the flow of glucose, over time, the concentration of the produced H_2_O_2_ enriches inside the immobilization section. When the flow is restarted, the accumulated H_2_O_2_ is flushed over the electrodes, resulting in an amplified current peak (Supplementary Figure S3). The current peak height was analysed for data analysis, as it depends on the amount of bound GOx, which is present in the channel, and thus, on the analyte concentration in the sample solution.

### System calibration

The designed ß-lactam bioassay was transferred from the microtiter plate to the presented microfluidic chip. Each step was adapted and optimized for on-chip measurements (Supplementary Figures S4, S5 and S6). Although, not a problem on microtiter plates, the adsorption of the PBP3 protein proved to be difficult on the microchannel surface. The adsorption of a protein to a surface involves different kinds of interactions (e.g. electrostatic, hydrophobic, etc.) and is thus, highly dependent on the protein and on the surface used. This phenomenon is therefore, hardly predictable. To solve this problem, an anti-His tag antibody, conveniently adsorbed on the chip surface (contrary to the PBP3), was introduced as capture element to the final on-chip setup, moreover, offering a better accessibility of the PBP3. Thus, the immobilization area was first coated with monoclonal anti His-tag antibodies. Afterwards, the surface was saturated with BSA to avoid unspecific binding before incubating the His-tagged PBP3 receptor. The sample was then mixed with the ampicillin-biotin conjugate and introduced to the channel. In the last step, the enzyme avidin-GOx was applied to the chip. Finally, the electrochemical readout of the assay was performed.

**Figure 2:**
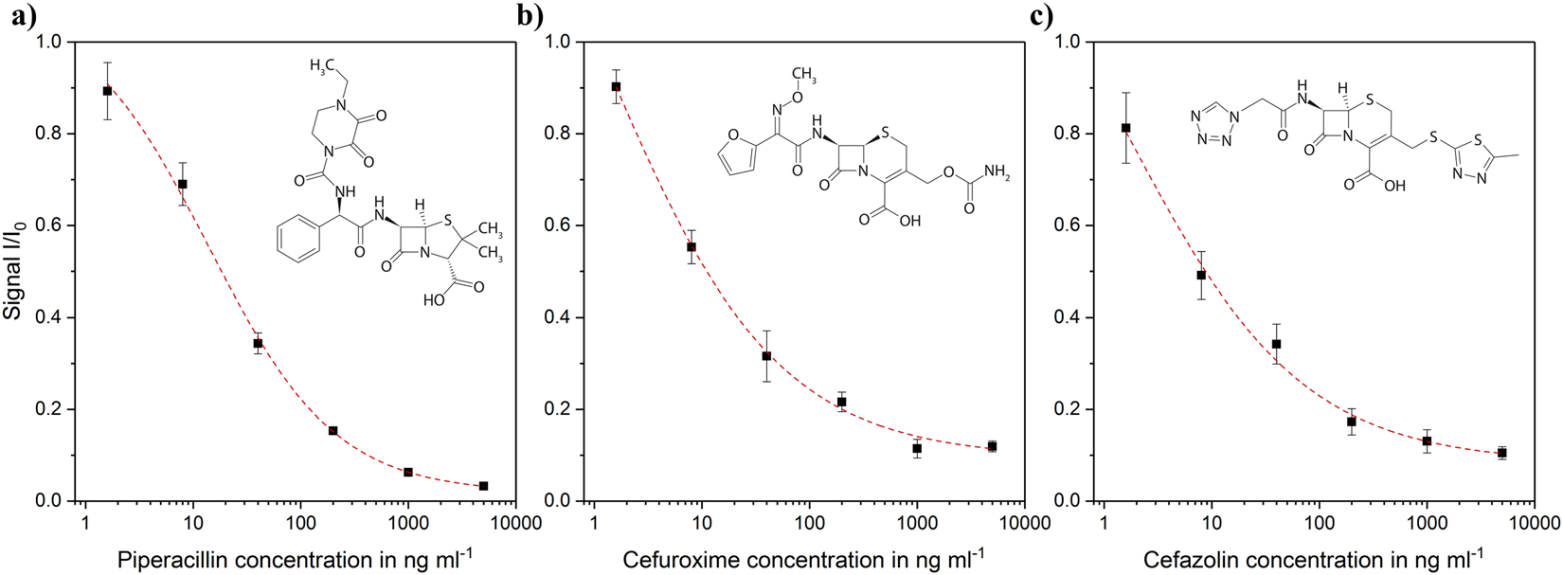
Calibration curves (n = 4) of (**a**) piperacillin, (**b**) cefuroxime and (**c**) cefazolin in human serum diluted 1:1000 in PBS for stopflow times of 2 min. The calibration points are fitted with a 4-parametric logistic curve, resulting in an inter-assay coefficient of variation of less than 20% and LODs of 2.07 ng ml^−1^, 4.88 ng ml^−1^ and 5.71 ng ml^−1^ for piperacillin, cefuroxime and cefazolin, respectively. I/I0 represents the current peak obtained for the measured sample (I) normalized to the current peak for a sample free of antibiotic (I)

For the quantification of different ß-lactam concentrations in clinically relevant specimens, like human serum, a calibration curve of each antibiotic of interest was required. Following the optimization of the assay parameters, the calibration curves of three antibiotics, two cephalosporins (cefuroxime and cefazolin) and one penicillin (piperacillin), were recorded. Therefore, solutions, containing different concentrations (1.6 to 5,000 ng ml^−1^) of piperacillin, cefuroxime and cefazolin, respectively, were prepared and applied to the functionalized channel of the biosensor. The results of the obtained calibration curves are presented in Fig. 2. The assays showed an adequate reproducibility with an inter-assay coefficient of variation (CV) of below 20% and a high sensitivity with limits of detection (LODs) of 2.07, 4.88 and 5.71 ng ml^−1^ for piperacillin, cefuroxime and cefazolin, respectively (Table 1).

**Table 1:** Parameters of the antibiotic assays, obtained from the 4-parametric fit of the respective calibration curve.

	Piperacillin	Cefuroxime	Cefazolin
**Fit quality**	0.9988	0.9954	0.9919
**Slope**	0.732	0.530	0.525
**Half maximal inhibitory concentration in ng ml** ^**−1**^	18.35	11.06	8.74
**Limit of detection in ng ml** ^**−1**^	2.07	4.88	5.71
**Limit of quantification in ng ml** ^**−1**^	6.28	14.78	17.29
**Inter-assay coefficient of variation in %**	≤15.9	≤17.9	≤19.5

### Clinical bedside monitoring of ß-lactams

**Figure 3:**
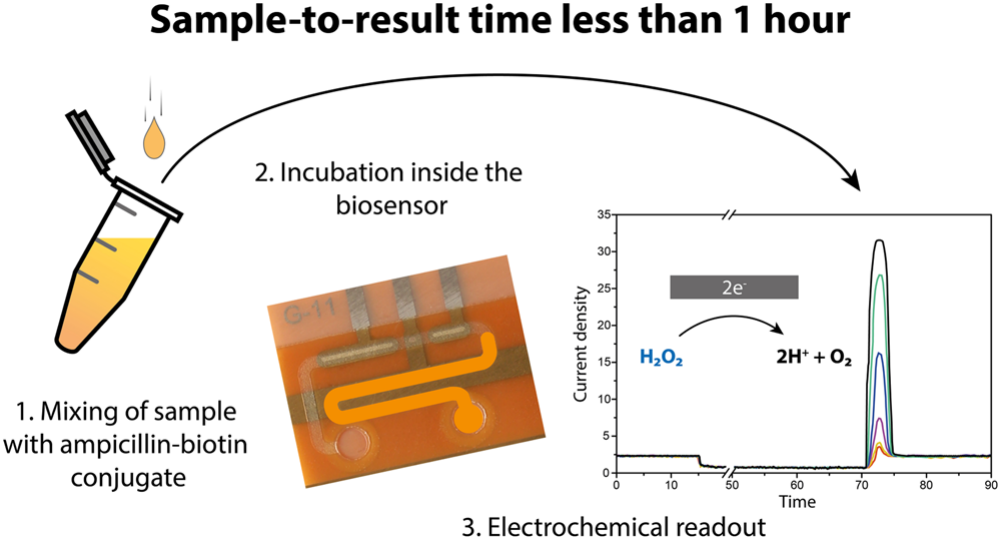
Illustration of a bedside monitoring scenario. After the sample is extracted from the patient, it is mixed with the ampicillin-biotin conjugate (1.) and incubated in the biosensor’s immobilization section (2.). After applying the avidin-GOx enzyme, the electrochemical read-out of the sensor completes the measurement (3.), resulting in an allover sample-to-result time of less than 1 hour.

The clinical measurements were performed on two patients undergoing antibiotherapy during surgery. The platform and measurement setup, including a syringe pump for applying a constant substrate flow and a laptop for the electrochemical readout, were placed in a room near the OR, simulating a bedside measurement situation (Supplementary Figure S7). In prospect of a bedside monitoring and for convenient handling, the biosensor chips were pre-functionalized the day prior to the planned measurement and stored at 4 °C. Both patients (patient 1: female, 60 kg and patient 2: male, 110 kg) received the usual recommended intravenous infusion dose for surgical prophylaxis: patient 1 received 1.5 g of cefuroxime and patient 2 2.0 g of cefazolin. Subsequently, blood specimens were up-taken from the vein at different time points to follow the patient’s personal pharmacokinetic. The initial sample was taken before the patient received their infusion of the antibiotic and served as a reference and positive control for the assay. For the measurement, after the centrifugation of the blood, 1 µl of the isolated plasma was diluted (1/500) with the ampicillin-biotin conjugate solution. The mixed sample was introduced for 30 minutes to the pre-functionalized channel. After a 15-minute incubation of avidin-GOx, the assay was electrochemically read out. This resulted in an overall sample-to-result time of less than 1 hour, perfectly compatible with therapeutic drug monitoring and fast adjustment of treatment (Fig. 3)^27^.

**Figure 4:**
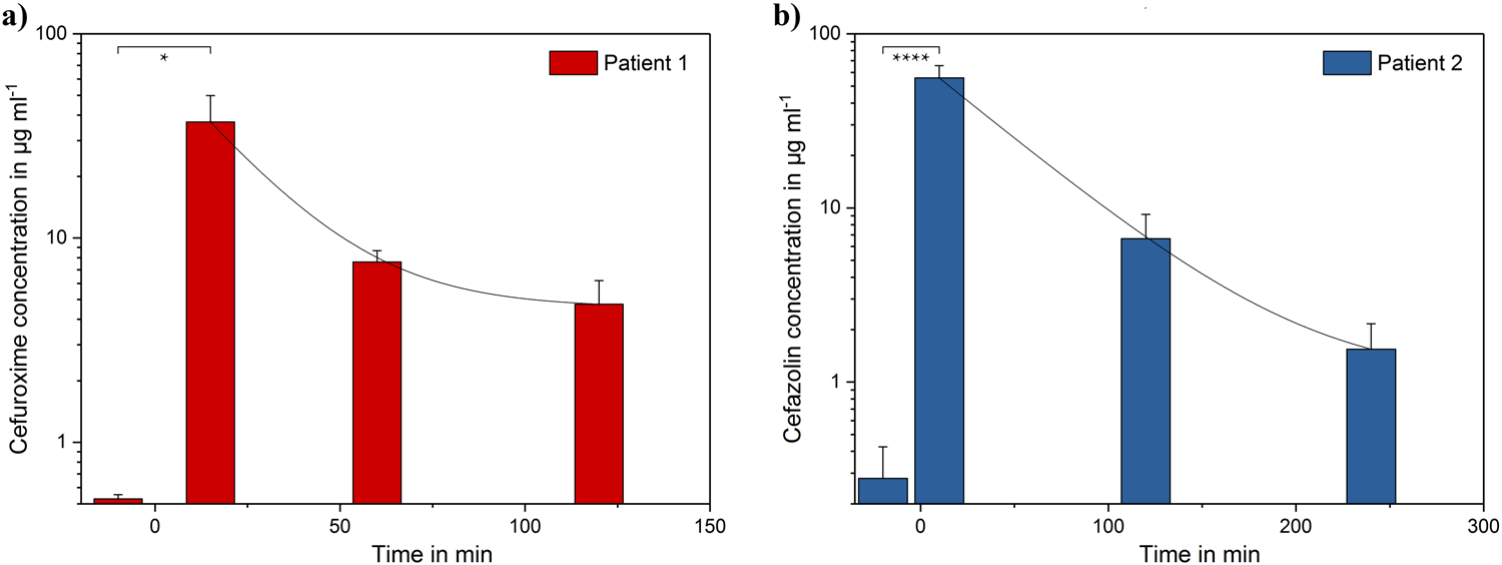
Clinical results of the on-site monitoring of two patients, undergoing surgery. The serum was diluted 1:500 in PBS and measured 4 times for each patient. The initial measurement was completed before the patients received their infusion (t = 0 min), the second 10 to 15 min after the infusion and the third and fourth measurement was performed after 90 and 180 min for cefuroxime and 120 and 240 min for cefazolin. (**a**) Patient 1 received cefuroxime, while patient 2 (**b**) received cefazolin as antibiotic. The data were analysed by an unpaired parametric two-tailed t-test (*P < 0.005, ****P < 0.0001).

Figure 4 presents the obtained results for the different patients and time points. For both patients, the initial measurement before the beginning of the antibiotherapy confirmed the absence of antibiotics in the plasma. 10 to 15 minutes after the patients received their infusion, the highest antibiotic concentrations were measured: 37.0 µg ml^−1^ for patient 1 and 55.7 µg ml^−1^ for patient 2. As the patient received the antibiotics intravenously, the maximal blood concentration C_max_ is typically reached immediately after the injection. Thus, C_max_ could not be measured and was therefore estimated by an exponential extrapolation to 72.9 and 76.3 µg ml^−1^ for patient 1 and 2, respectively.

First, the measured concentrations were rapidly decreasing, due to the distribution of the antibiotics in the tissues and the whole body. Some pharmacokinetic parameters of interest were determined from the measurements: the volume of distribution V_D_ was 13.8 and 5.2 L for cefuroxime and cefazolin, respectively, the clearance CL of the antibiotics was 7.2 L h^−1^ for cefuroxime and 2.8 L h^−1^ for cefazolin and the half-life time of both antibiotics was found to be 1.3 h (Table 2).

**Table 2:** Calculated pharmacokinetic parameters of patient 1 and 2.

	Patient 1 – Cefuroxime	Patient 2 – Cefazolin
	Female, 60 kg	Male, 110 kg
**Initial dose in g**	1.5	2.0
**Estimated maximal concentration in µg ml** ^**−1**^	72.9	76.3
**Half-life time in h**	1.3	1.3
**Plasma protein binding in %**	33^28–30^	80^31, 32^
**Volume of distribution in L**	13.8	5.2
**Clearance in L h** ^**−1**^	7.2	2.8
**Time above 8 µg ml** ^**−1**^ **in min**	60	111

## Discussion

Therapeutic drug monitoring is the first step towards a personalized therapy. Many therapies would benefit from a therapeutic drug monitoring, especially for drugs with narrow therapeutic windows, with marked pharmacokinetic variability or known to cause important adverse effects. To date, the benefits of a systematic monitoring program for antimicrobial compounds have been demonstrated for aminoglycosides only^33^. However, the decreasing susceptibility of bacteria to available antimicrobials, as well as some clearly emerging data on pharmacokinetic variability suggests that the advantages could be much higher. The challenge to develop such programs mainly lies in the development of rapid bioassays that can accurately determine antimicrobial concentrations in blood or other specimens. Conventionally, the measurement of antibiotics is performed in a central laboratory via microbiological tests based on bacterial growth inhibition. Regardless of their low-cost and simple handling, they are time-consuming and cannot be performed at the bedside. Existing alternative state of the art techniques such as liquid chromatography (LC), also combined with mass spectrometry (LC−MS)^34^ or UV (LC−UV)^35^ detection, and capillary electrophoresis (CE) are expensive, need extensive sample preparation, are therefore time intensive and have to be executed by specialized personnel in specific external laboratories^36^. Immunoassays, which were initially designed for laboratory use, can also be adapted for POCT. But the majority of them rely on antibodies for the detection of a specific antibiotic^37^. However, antibodies are not always available and often undergo batch to batch variations and lack of stability^38^.

In this work, we present a platform for bedside therapeutic drug monitoring, which aims to overcome the technical limitations encountered with the currently available analytical methods. The proposed miniaturized microfluidic system provides a practical and functional solution with reduced reagent and sample consumption (580 nl), combined with a fast and easy to handle bioassay. It enables short sample-to-result times (less than 1 hour) and offers great possibilities for future adaptation and expansion for the detection of additional drugs or biomarkers of interest.

Here, we focused on the monitoring of ß-lactam antibiotics, a broad-spectrum class of antibiotics, named after the ß-lactam ring in their chemical structure and frequently used for the prophylaxis and treatment of infectious diseases in human medicine. We aimed at designing a single bioassay that would be able to detect this class of antibiotics, avoiding implementing a bioassay for each specific antibiotic of this class. Generating specific antibodies against the ß-lactam ring seems to remain the bottleneck for the development of such an immunoassay, due to the *in-vitro* instability and chemical reactivity of antibodies. Typically, antibodies against penicillins, which are described so far, are either highly specific or have limited group specificity for a few closely related compounds^39, 40^. To tackle this issue, we took advantage of one target of ß-lactam antibiotics, the PBP3 from *S. pneumoniae*, which is generic as well as highly specific towards the active form of ß-lactams. Therefore, as an alternative to antibodies, this naturally occurring receptor appeared to be very advantageous in regards of sensitivity and specificity.

It has been well described that the pharmacokinetic of ß-lactam antibiotics strongly differ in critically ill or obese patients, as well as in those with renal dysfunction^41, 42^. A study in an ICU estimated that 73% of patients fell outside the desired pharmacokinetic/pharmacodynamic range and implied that ß-lactam monitoring would in these cases be useful and necessary^42^. Clinicians working with such patients can have a hard time choosing an antimicrobial dose that will confidently achieve a target drug exposure. In this work, we successfully demonstrated the applicability of our electrochemical, microfluidic platform for the therapeutic drug monitoring of ß-lactams via the bedside measurement of the individual pharmacokinetic of two patients undergoing surgery.

For both antibiotics, the minimal inhibitory concentrations (MIC) for most common pathogens, such as *E. coli* or *S. aureus*, are reported to be close to 8 µg ml^−1^ ^43^. As ß-lactams show time-dependent killing, the longer bacteria are exposed to concentrations above the MIC, the higher the killing efficacy^44^. Thus, it is common medical practice to target a maximal concentration equal to 4 or 5 times the MIC, so that the antibiotic concentration remains above the MIC during 50% of the treatment interval and to achieve maximal protection^44^. In our study, the measured blood concentrations of active antibiotics for both patients were indeed above 8 µg ml^−1^ for 60 to 111 min, which represents more than 50% of the surgical procedure of 2 to 3 h.

Intravenous infusion of ß-lactam antibiotics is reported to follow a two-compartment kinetic model^45^; once the distribution phase is over, the measured concentrations are decreasing slowly over time due to the metabolization and renal clearance of the antibiotics. The two-compartment model could be observed on both patients, whereas the process of the clearance of the antibiotics were, as expected, not identical. A direct comparison of the pharmacokinetic parameters between the two patients is hardly possible. Given the fact that they received different treatments (different cephalosporins and dose), behaving in a different way in the body (e.g. different percentage in plasma protein binding), the patients’ response to the antibiotherapy also depends on numerous other different conditions (e.g. gender, weight, pathological condition), which makes a personalized antibiotherapy essential. Additionally, patient 1 suffered from diabetes mellitus type 2, which could also influence the pharmacokinetic response^46^. Hence, the calculated pharmacokinetic parameters could only be compared to previously reported ones for healthy subjects, and were globally in the same range.

The reported serum half-lives were 1.2–1.8 h for cefazolin and 1.1 h for cefuroxime^47^, fitting to the 1.3 h calculated in our study, but can also vary over a wide range, depending on the pathological status of the patient^48^. One important pharmacokinetic parameter is the apparent volume of distribution, which reflects the distribution of the drug within the body. It is determined from the injected dose and the maximum plasma drug concentration measured immediately after infusion. In certain pathologies (e.g. altered fluid status, change in serum albumin concentrations, etc.) the volume of distribution of the drug is altered. In such cases, the dosing of the drug must be adapted, as a standard treatment may not reach the expected blood level for proper efficacy. The here measured and calculated values for the total amount of bound and unbound antibiotics lead to a volume of distribution of 13.8 and 5.2 L for cefuroxime and cefazolin, respectively, which is in the range of the reported data in the literature^28, 29, 49^. The clearance, a measure of drug elimination from the body, and is based on the half-life time and the volume of distribution. It generally reflects the hepatic and renal function of the patient. This parameter is mainly of interest for drugs with high potential toxicity, as a decreased clearance may lead to a prolonged exposure to high drug level. For cefuroxime, a clearance of 7.2 and for cefazolin a clearance of 2.8 L h^−1^ was calculated. The stated data in the literature is around 8.0 L h^−1^ for cefuroxime^50^, and in the range of 3.0 L h^−1^ for cefazolin^51, 52^, which fits well with our results.

To conclude, given the strong association between inappropriate antibiotherapy in infected critically ill patients and mortality^53^, the here proposed biosensor for therapeutic bedside monitoring is applicable for the fast and low-cost quantification of ß-lactam antibiotics in plasma samples. This could pave the way to a personalized antibiotherapy, reducing improper over- and under-dosing, resulting in an improved outcome of the patient and will further decrease in long-term perspective the development of multidrug-resistant bacteria. Following studies, focusing among other things on the dose dependant pharmacokinetics of ß-lactams, and performed on cohorts, including different patients with various pathologies, will enable the quantitative assessment of the usefulness of a personalized therapy with this new technique.

## Methods

### Chip design and fabrication

The immobilization section with a volume of 580 nl and a surface-to-volume ratio of 155 cm^−1^ allows a defined implementation of the protein-based assay by capillary filling of the channel. The removal of excessive assay reagents is realized through the inlet by applying a vacuum, which ensures that no biomolecules get in contact with the electrodes. After each immobilization step, the channel is flushed with 50 µl of wash buffer (0.01 M PBS with 0.05% TWEEN^®^ 20) to remove all unbound reagents.

**Figure 5:**
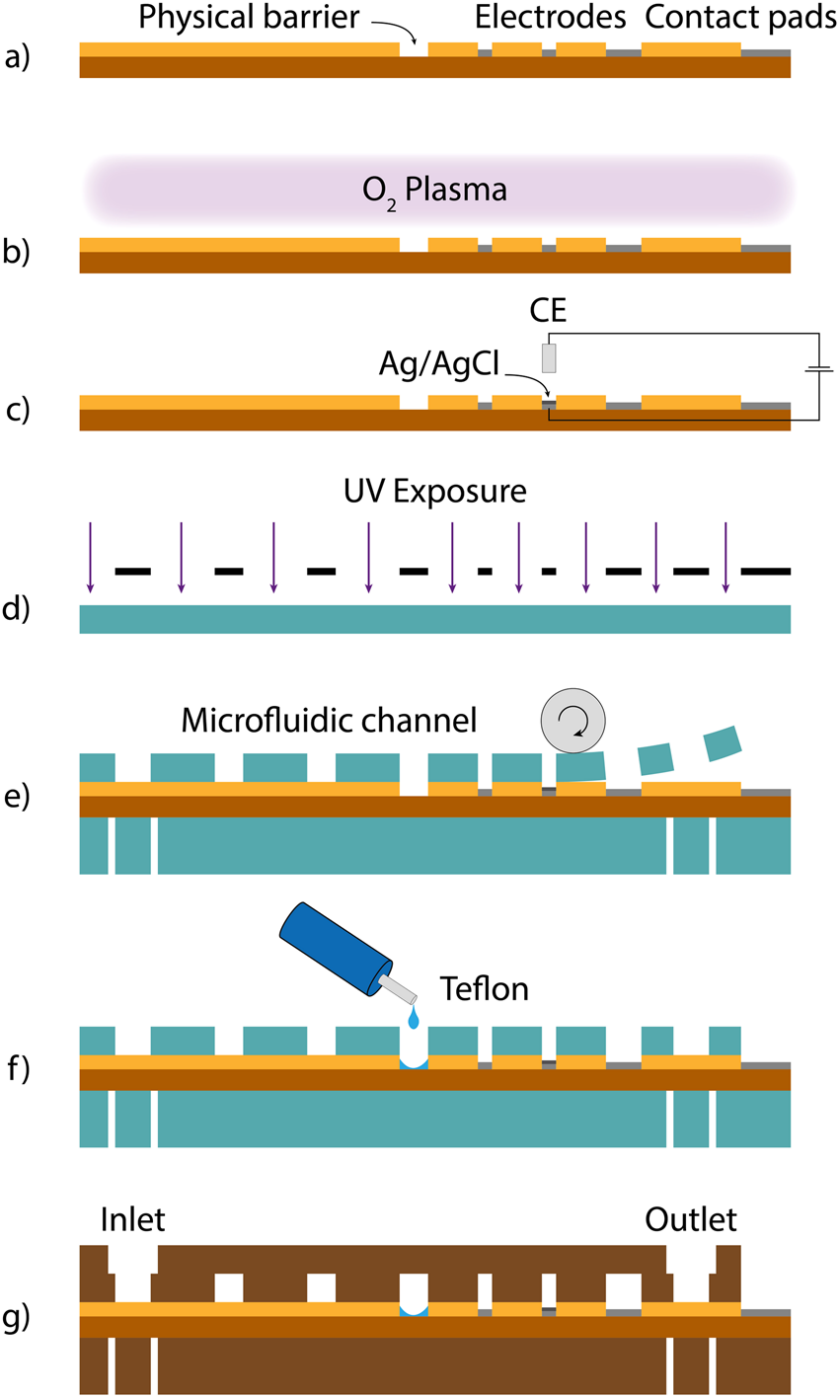
Illustration of the different fabrication steps of the microfluidic biosensor platform: (**a**) a Pt structured polyimide substrate isolated with SU-8 (**b**) O_2_ Plasma process to remove SU-8 residues on the Pt electrodes (**c**) galvanic deposition of the Ag/AgCl reference electrode (**d**) exposure of different DFR layers (**e**) lamination of the DFR layers onto the substrate (**f**) Applying Teflon^®^ to embed the stopping barrier (**g**) final electrochemical microfluidic biosensor

To keep the disposable biosensor as cheap as possible, the fabrication process of the microfluidic platform mainly relies on low-cost materials, like polymers, to create the channel. In this sense, the DFR technology offers the best way to rapidly manufacture new structures in an inexpensive way without the need of a clean room process. The fabrication procedure of the chip, as described in our previously published work^18^, was further simplified, which results in a shorter overall manufacture time of the chip. The overall manufacture process is illustrated in Fig. 5.

As a substrate of the biosensor, Pyralux^®^ AP8525R (DuPont) is used after 1 hour of copper etching. The electrodes are realized, using lift-off technology. The resist MA-N 1420 (Micro Resist Technology) is spin coated onto the substrate at 3,000 rpm for 30 s, exposed to UV light and developed. Afterwards, in a physical vapour deposition step, the only clean room process, a 200 nm thick Pt layer is realized on the substrate, creating the electrodes. To precisely define the electrode areas, to electrically isolate the electrodes and to realize small wells for the stopping barrier, a 5 µm thick SU-8 3005 (MicroChem Corp.) layer is spin coated on the substrate at 4,000 rpm for 30 s, after removing the resist MA-N 1420. After UV exposure and developing, the wafer is hard baked in an oven for 1 hour at 150 °C.

In order to remove SU-8 residues on the Pt electrodes, a plasma step is induced. The low frequency oxygen plasma is applied for 1:30 min at 200 W at room temperature (Tetra-30-LF-PC, Diener).

As a reference electrode, an Ag/AgCl on chip electrode is realized by galvanic deposition. First, all contact pads are passivated with an UV sensitive tape (1020 R, Ultron Systems Inc.). The galvanic silver deposition is then performed in an Arguna S solution (Umicore Galvanotechnik), employing a current density of −4.5 mA cm^2^ for 10 min with a silver counter electrode. The silver layer is then chlorinated in a 0.1 M KCl solution at +0.6 mA cm^−2^ for 7.5 min, using a Pt counter electrode.

To implement the 500 µm width channels, different exposed 63.5 µm thick Pyralux^®^ PC1025 (DuPont) DFR layers are stacked on the substrate. The DFR layers are first exposed for 2:30 min to UV light on an exposure unit (Hellas, Bungard Elektronik) and then developed in an ultrasonic bath with a 1% sodium carbonate (Na_2_CO_3_) solution at 42 °C. To stop the development, the DFR layers are exposed to a 1% HCl bath for 2 min. After the development, the layers are aligned on the substrate and then fixed onto the substrate using a laminator (HRL 350, Ozatec).

In order to realize a proper hydrophobic stopping barrier for the separation of the immobilization area and the electrochemical cell, a small drop of 1% Teflon^®^ (AF 1600, DuPont) is dispensed into the small SU-8 wells by a hand dispenser. After employing the stopping barrier, the channels of the biosensor are sealed with another DFR layer. To prevent bending of the wafer, two DFR layers are stacked on the backside of the substrate. In the last step, the wafer is diced into the individual biosensors with an ordinary pair of scissor and is then hard baked at 160 °C for 3 hours. All manufacturing steps combined, the total fabrication time amounts to roughly 10 h, while the costs add up to less than 0.50 € per single biosensor (Supplementary Table S8).

### Production and purification of the penicillin-binding protein 3 (PBP3)

A synthetic version of the PBP3* gene from *Streptococcus pneumoniae* R6 was ordered as G-block at IDT (Integrated DNA technologies Inc.). This gene encodes a truncated form of the PBP3 (Gly15 to Lys394) protein with an N-terminal hexa-histidine tag for purification. The gene was Gibson assembled into the pRSETmod^16^ plasmid allowing for bacterial expression under control of the T7 promoter. *E. coli* BL21* (DE3) pLysS were transformed with the expression vector, cells were grown in LB medium (Luria/Miller, Carl Roth, X968) supplemented with ampicillin (100 µg ml^−1^) and chloramphenicol (34 µg ml^−1^) at 37 °C until OD600 = 0.6. Protein production was then induced with 1 mM β-D-1-thiogalactopyranoside (IPTG) for 14 h at 20 °C. Cells were harvested by centrifugation (6,000 g, 10 min at RT), resuspended in lysis buffer (35 ml per 1000 ml initial culture volume, 50 mM NaH_2_PO_4_, 300 mM NaCl, 10 mM imidazole pH 8.0), freeze-chocked and lysed by sonication (Bandelin, 60%, pulse 0.5 s every s for 10 min). Subsequently, cells debris were eliminated by centrifugation (30,000 g, 30 min, 4 °C). Proteins were purified from the supernatant on a gravity flow Ni^2+^-NTA-agarose Superflow column (Qiagen, Hilden, Germany, cat. no. 30210) following manufacturer instructions. Protein purity was analysed by sodium dodecyl sulphate–polyacrylamide gel electrophoresis (SDS–PAGE) and the protein concentration was determined by the Bradford method (Bio-Rad, Hercules, CA, cat. no. 500–0006) using bovine serum albumin (BSA) as standard. Proteins were diluted to 1 mg ml^−1^ in elution buffer (50 mM NaH_2_PO_4_, 300 mM NaCl, 250 mM imidazole pH 8.0) containing 50% glycerol and stored at −20 °C.

### Synthesis of the ampicillin-biotin conjugate

Three ampicillin-biotin conjugates with increasing linker lengths between the ampicillin and the biotin were synthesized: ampicillin-biotin (conjugate 1), ampicillin-PEG12-biotin (conjugate 2) and ampicillin-PEG24-biotin (conjugate 3). Ampicillin (Carl Roth K029) was covalently linked to biotin via an amide bond, using different amino-reactive biotin derivates (conjugate 1: EZ-link NHS-LC-Biotin, Thermo Scientific cat. no. 21336; conjugate 2: EZ-link NHS-PEG12-Biotin, Thermo Scientific cat. no. 21312; conjugate 3: EZ-link NHS-PEG24-Biotin, Pure-PEG cat. no. 246924–50). A two-time molar excess of biotin derivate was reacted with ampicillin (final concentration 1 mg ml^−1^) in water free ethanol for one hour at room temperature under constant shaking. More precisely, for conjugate 3: a 50 mg ml^−1^ stock solution of EZ-link NHS-PEG24-Biotin was prepared in DMSO. 1 mg ampicillin were dissolved in 842 µl ethanol and mixed with 158 µl of the EZ-link NHS-PEG24-Biotin stock solution, resulting in a final volume of 1 ml containing 2.5 µmol ampicillin and 5 µmol EZ-link NHS-PEG24-Biotin. The coupling reaction was followed by thin-layer chromatography (TLC), using a silica gel (Fluka, 91835-50EA) as stationary phase and a solution of acetone-acetic acid (95:5) as solvent. The reaction products migrated at an R_f_ value of 0.697 and the ampicillin appeared to be completely reacted. The conjugates were aliquoted and stored at −20 °C.

### Assay incubation procedure

All assay components and proteins were diluted in 10 mM phosphate buffered saline (PBS) solution, pH 7.4, containing 0.137 M NaCl and 2.7 mM KCL. Human serum samples for the calibration curves were obtained from the University Medical Center Freiburg. The immobilization capillary was first coated with 200 µg ml^−1^ monoclonal anti His-tag antibodies (VWR, NOVG70796-3) for one hour at room temperature. The surface was then saturated with 1% BSA, before the incubation with 5 µg ml^−1^ His-tagged PBP3 at 4 °C overnight. The sample, mixed with the ampicillin-biotin conjugate (40 ng ml^−1^), was introduced for 30 minutes to the functionalized channel and then 1 µg ml^−1^ Avidin-GOx was added for 15 minutes. Between each incubation step, any unbound reagents and biomolecules were removed by washing with 50 μl of wash buffer (PBS containing 0.05% TWEEN^®^ 20). A 40 mM glucose solution in 0.1 M PBS was used as substrate for the readout of the glucose oxidase enzyme linked assay. The 4 channel potentiostat (EmSTAT3, Palms Sens, Netherlands), controlled by the delivered software (MultiTrace 3.5), was set to constant polarization mode for the electrochemical readout. During the measurement, a constant flow rate of 20 µl min^−1^ was applied by a syringe pump (PHD Ultra, Harvard Apparatus, USA). Preliminary to the measurement, a short pre-treatment of the working electrode was executed. The working electrode was polarized for 5 s to 0.8 and −0.05 V vs. the on-chip reference electrode for 30 cycles. Furthermore, the working electrode was oxidized at 0.8 V for 60 s to enhance the signal stability and the sensitivity to H_2_O_2_.

### Statistical analysis

All data are expressed as mean ± standard error with n = 4. For calculating the limit of detection (LOD) and the limit of quantification (LOQ), the standard error of the highest concentration (i.e. 5,000 ng ml^−1^) of the calibration curve is multiplied by 3.3 or 10, respectively, and is then divided by the slope of the fitted 4 parametric logistic curve. Differences between the absence of the antibiotic and the highest measured value are analysed by an unpaired parametric two-tailed t-test. For calculating the half-life time (t_1/2_), the second part of the two-compartment model is used to determine the time until the blood concentration drops to the half. The volume of distribution (V_D_) is determined by the initial dose of the antibiotic, which the patient received, divided by the estimated blood concentration of all bound and unbound antibiotics. In order to obtain the clearance (C_L_) value of the patient, V_D_ is multiplied with ln(2) and divided by the half-life time.

## Electronic supplementary material

Supplementary information
