# Supplementary material for: Clinical on-site monitoring of ß-lactam antibiotics for a personalized antibiotherapy
Source: Sci Rep. 2017 Jun 9;7:3127. doi: 10.1038/s41598-017-03338-z (PMC5466632; doi:10.1038/s41598-017-03338-z)
Supplement: Supplementary file 1 — Supplementary information [file 41598_2017_3338_MOESM1_ESM.pdf]

Supplement information

Clinical on-site monitoring of ß-lactam antibiotics for a personalized antibiotherapy

R. Bruch[1†], C. Chatelle[2, 6†], A. Kling[3], B. Rebmann[2, 6], S. Wirth[4], S. Schumann[4], W. Weber[2, 6], C. Dincer[1,5*], and G. Urban[1,5]

[1]Department of Microsystems Engineering, University of Freiburg, 79110 Freiburg, Germany
[2]Faculty of Biology, University of Freiburg, 79104 Freiburg, Germany
[3]Department of Biosystems Science and Engineering, ETH Zurich, 4058 Basel, Switzerland
[4]Department of Anaesthesiology and Critical Care, Medical Center – University of Freiburg, Faculty of Medicine, University of Freiburg, 79106 Freiburg, Germany
[5]Freiburg Materials Research Center, University of Freiburg, 79104 Freiburg, Germany
[6]BIOSS Centre for Biological Signalling Studies, University of Freiburg, 79104 Freiburg, Germany

[†]These authors contributed equally to this work
[*]dincer@imtek.de

Performance test of the synthesized conjugates

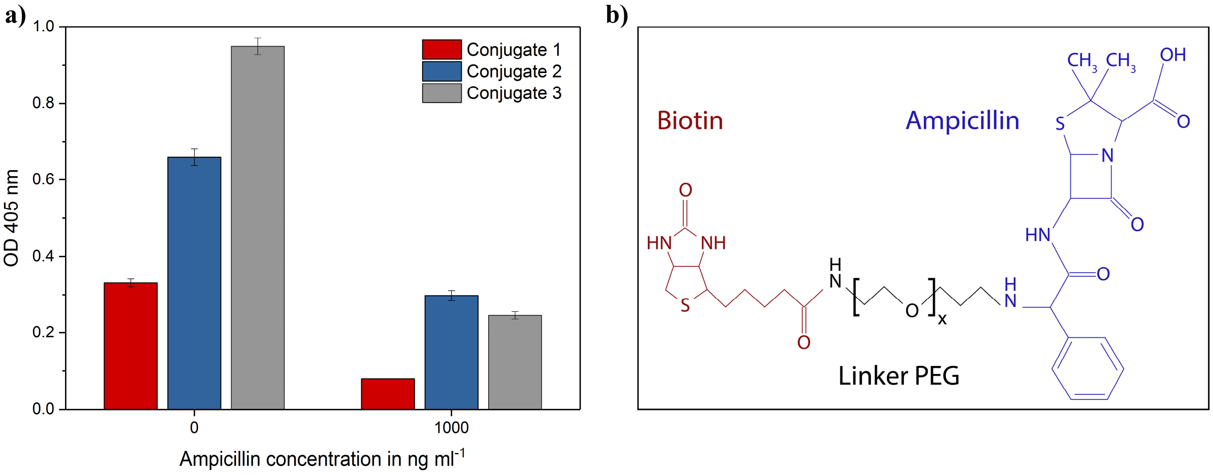

S1: a) Comparison of the optical results obtained using the three synthesized conjugates with different linker lengths, tested on a microtiter plate (n=4) for an ampicillin concentration of 0 and 1000 ng ml^{-1} after 30 min of enzymatic reaction and a conjugate concentration of 500 ng ml^{-1}. Conjugate 1: ampicillin-biotin, conjugate 2: ampicillin-PEG12-biotin and conjugate 3: ampicillin-PEG24-biotin. b) Chemical structure of the different conjugates, where x equals 0, 12 and 24 for the conjugate 1, 2 and 3, respectively.

Proof of principle testing of the designed bioassay on a microtiter plate

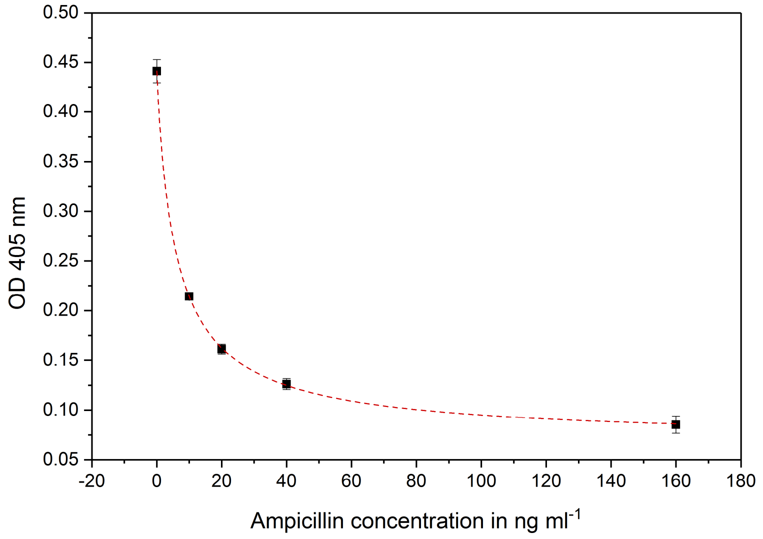

S2: Proof of principle and feasibility of the designed bioassay: Calibration curve of ampicillin performed on a microtiter plate (Costar cat. no. 3590) and optically read-out. 10 µg ml^{-1} of PBP3 in 50 mM bicarbonate buffer pH 9.6 was coated overnight at 4 °C on the surface of the microtiter plate. After a 1 h blocking step (300 µl of 1 % BSA in PBS buffer), the sample mixed with conjugate was added and incubated for 1 h, followed by 100 µl of 1 µg ml^{-1} avidin-GOx for 30 min. Finally 100 µl of substrate solution (PBS buffer containing, 40 mM glucose, 375 µg ml^{-1} ABTS and 40 ng ml^{-1} HRP) was added to the well. The enzymatic reaction was followed via the measurement of product formation by spectrophotometry at 405 nm for 30 minutes.

Stop-flow measurement technique

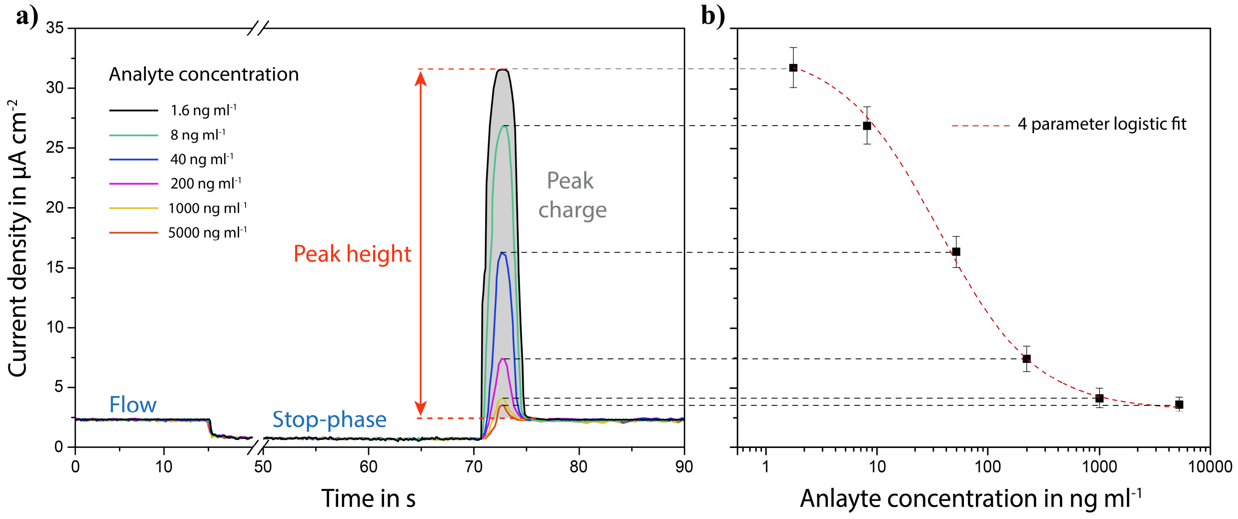

S3: Illustration of the applied stop-flow protocol for 6 different analyte concentrations. a) A flow of 40 mM glucose solution at a rate of 20 µl min^{-1} was applied. During the stop-phase (1, 2 or 5 min), the enzyme's production of H_2O_2 goes further on. By restarting the flow, the accumulated H_2O_2 is flushed through the electrochemical cell, where the hydrogen peroxide is electrochemically detected, resulting in an on-chip calibration curve b).

Optimization of the blocking method and time

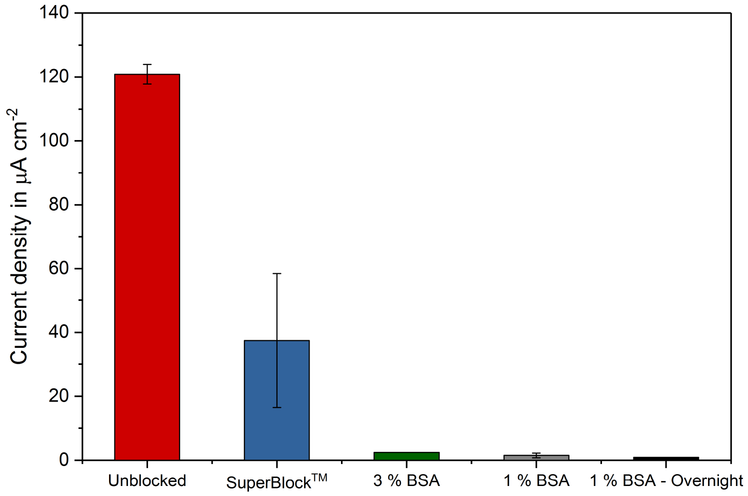

S4: Comparison of different blocking strategies after 15 min immobilization of 1 µg ml^{-1} GOx labeled avidin, followed by a 1 min stop-flow readout technique with 40 mM glucose solution. All blocking methods, except the overnight blocking, were applied for 1 hour. The best blocking efficiency for glucose oxidase labeled avidin was achieved with overnight blocking of 1 % BSA solution. However, an incubation time of 1 hour was used for the assay protocol, since there was no significant improvement of the blocking efficacy compared to the overnight blocking procedure. The error bars show the standard deviation of two parallel measurements.

Performance characteristics of the microfluidic platform

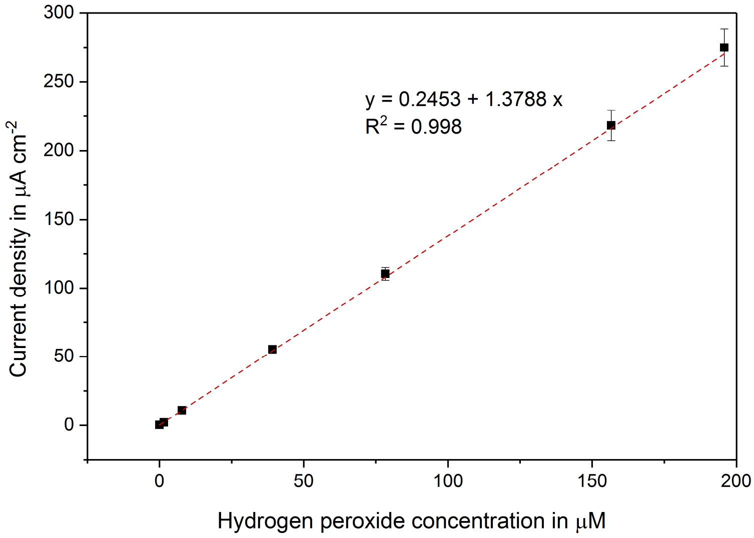

S5: Calibration curve (n=8) of hydrogen peroxide by amperometric detection, using different hydrogen peroxide solutions varying from 1.56 to 195.8 µM, prepared from a 30% (w/w) stock solution. A constant flow rate of 20 µl min^{-1} was applied during the measurement at a working electrode polarized to 0.45 V vs. the on-chip Ag/AgCl reference electrode.

Glucose substrate concentration dependency

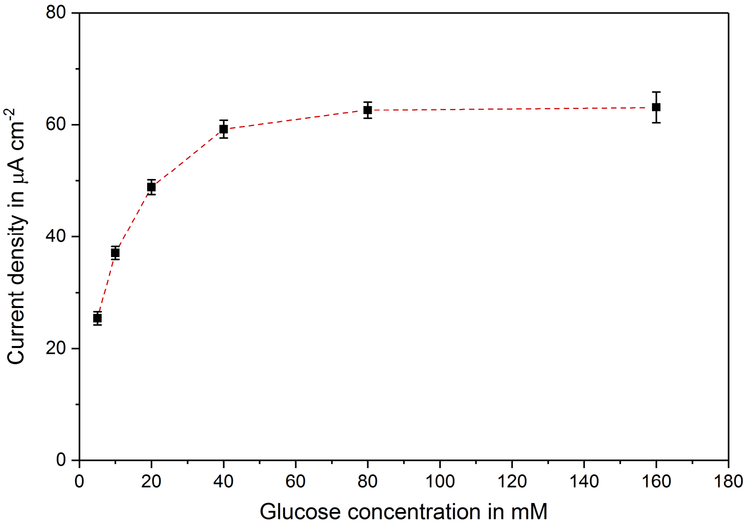

S6: Substrate concentration dependency of the stop-flow signals, measured with different glucose solutions, varying from 5 to 160 mM in 0.1 M PBS solution. Using a GOx labeled avidin assay with a glucose solution flow rate of 20 µl min^{-1}, an amperometric 2 min stop-flow signal readout is performed. The error bars show the standard deviation of 4 parallel measurements.

Measurement setup

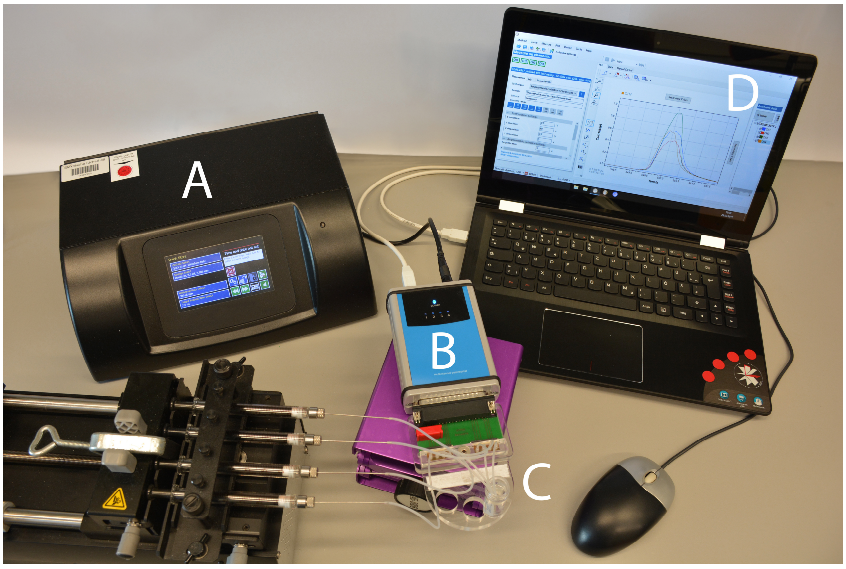

S 7: Photograph of the overall measurement setup, including a syringe pump to apply a constant flow (A), a 4-channel potentiostat for the amperometric measurement (B), a custom-made chip holder to realize the fluidic and electric connection of the chip (C), and a laptop for the signal readout (D).

Estimation of cost and time-to-fabricate

To estimate the costs per single biosensor, the calculations are based on the fabrication of 460 wafers. For the total cost of a single sensor, each material and fabrication step is considered, including the costs for the polyimide substrate, the platinum pattern, the DFR layers and the chemical reagents like SU-8, silver and silver chloride electrolytes as well as developer and remover solutions. The estimated material costs per single chip are listed in table S8.

S8: Estimated costs of the fabrication materials per single biosensor.

Materials	Costs per single biosensor in €
Substrate – Pyralux® AP	0.10
ma-N resist	0.03
Pt pattern	0.25
SU-8	0.04
Solution for silver deposition	0.01
DFR – Pyralux® PC	0.03
Developer and Remover	0.03
**Total device costs**	**0.49**

Due to the low reagent consumption of the biosensor of less than 1 µl, the needed volumes for the immunoassay are also very low. For the coating of the surface with antibodies, a concentration of 200 µg ml^{-1} is used. Therefore, roughly 1250 biosensors can be coated with a stock antibody solution of 125 µl (at 2 mg ml^{-1}). Since the antibody solution is priced at about 600 € per mg, the antibody costs per single chip 0.12 €. Using similar calculations for the other used biomolecules, wash buffer and 1 % BSA blocking solution, the total costs for all reagents per single chip are about 0.13 €. Thus, the overall costs of the microfluidic device for the personalized antibiotherapy is calculated to be only 0.62 € per sample.

For the fabrication process of the DFR based biosensor, a total work time of 10 hours (excluding the Pt pattern and the hard bake times in the oven) is estimated. By terms of automation, the work time can be reduced, regarding the degree of automation of the process. Furthermore, the assay preparation, for example the antibody immobilization, can be even further more automated, since now every reagent is dispensed by hand. However, it is possible to combine the here employed system with a robotic technique for spotting multiple biomolecules on individual chip inlets in parallel, which increases the throughput and reduces therefore the chip preparation time.
